# HCS-Neurons: identifying phenotypic changes in multi-neuron images upon drug treatments of high-content screening

**DOI:** 10.1186/1471-2105-14-S16-S12

**Published:** 2013-10-22

**Authors:** Phasit Charoenkwan, Eric Hwang, Robert W Cutler, Hua-Chin Lee, Li-Wei Ko, Hui-Ling Huang, Shinn-Ying Ho

**Affiliations:** 1 Institute of Bioinformatics and Systems Biology, National Chiao Tung University, Hsinchu, 300, Taiwan; 2 Department of Biological Science and Technology, National Chiao Tung University, Hsinchu, 300, Taiwan; 3 Institute of Molecular Medicine and Bioengineering, National Chiao Tung University, Hsinchu, 300, Taiwan; 4 Program in Physics, School of Pure and Applied Sciences, Edison State College, Florida, 33919, USA

## Abstract

**Background:**

High-content screening (HCS) has become a powerful tool for drug discovery. However, the discovery of drugs targeting neurons is still hampered by the inability to accurately identify and quantify the phenotypic changes of multiple neurons in a single image (named multi-neuron image) of a high-content screen. Therefore, it is desirable to develop an automated image analysis method for analyzing multi-neuron images.

**Results:**

We propose an automated analysis method with novel descriptors of neuromorphology features for analyzing HCS-based multi-neuron images, called HCS-neurons. To observe multiple phenotypic changes of neurons, we propose two kinds of descriptors which are neuron feature descriptor (NFD) of 13 neuromorphology features, e.g., neurite length, and generic feature descriptors (GFDs), e.g., Haralick texture. HCS-neurons can 1) automatically extract all quantitative phenotype features in both NFD and GFDs, 2) identify statistically significant phenotypic changes upon drug treatments using ANOVA and regression analysis, and 3) generate an accurate classifier to group neurons treated by different drug concentrations using support vector machine and an intelligent feature selection method. To evaluate HCS-neurons, we treated P19 neurons with nocodazole (a microtubule depolymerizing drug which has been shown to impair neurite development) at six concentrations ranging from 0 to 1000 ng/mL. The experimental results show that all the 13 features of NFD have statistically significant difference with respect to changes in various levels of nocodazole drug concentrations (NDC) and the phenotypic changes of neurites were consistent to the known effect of nocodazole in promoting neurite retraction. Three identified features, total neurite length, average neurite length, and average neurite area were able to achieve an independent test accuracy of 90.28% for the six-dosage classification problem. This NFD module and neuron image datasets are provided as a freely downloadable MatLab project at http://iclab.life.nctu.edu.tw/HCS-Neurons.

**Conclusions:**

Few automatic methods focus on analyzing multi-neuron images collected from HCS used in drug discovery. We provided an automatic HCS-based method for generating accurate classifiers to classify neurons based on their phenotypic changes upon drug treatments. The proposed HCS-neurons method is helpful in identifying and classifying chemical or biological molecules that alter the morphology of a group of neurons in HCS.

## Background

To investigate the organization of neurons in various brain tissues including their activity and function, scientists typically examine neural images to classify distinct neuron morphologies [1]. In high-content screening (HCS), automated image analysis has become necessary to identify interesting samples and extract quantitative information by microscopy [2]. For rare phenotypes that are nonetheless recognizable by eyes, a researcher can generate a classifier to recognize cells with the phenotype of interest [2]. Recently, HCS-based methods have been used to quantify neuronal phenotypic changes which correlate to multiple treatments or drugs as illustrated in Table 1. Previously, the single-neuron neuromorphology was considered difficult because of tightly packed positioning and huge spanning arbors of neurons [1], [3]. However, the variation of neuronal morphology to a treatment effect should be considered as a global phenotypic change affecting a large number of neurons rather than only one specific neuron. The image containing multiple neurons is named a multi-neuron image. Thus, the multi-neuron based HCS plays a crucial role for drug treatment analysis [3-10]. In this study [8], the appropriate medication for Huntington's disease was identified.

**Table 1:** Methodologies for drug analysis of HCS neuron images since 2010

*Reference (year) *	*Automatic feature extrction*	*Multineuron supporting*	*Classification analysis*	*Regression analysis*	*Type of features^1^*	*Features extraction software *
[11] (2010)	No	No	No	No	snb	Free
[4] (2010)	Yes	Yes	No	No	snbc	Commercial
[5] (2010)	Yes	Yes	No	No	sn	Unavailable
[12] (2010)	Yes	No	No	No	n	Free
[13] (2010)	No	No	No	No	nb	Free
[6] (2011)	Yes	Yes	No	No	snbc	Free
[7] (2011)	Yes	Yes	No	No	snc	Free
[3] (2011)	Yes	Yes	No	No	snc	Free
[8] (2011)	Yes	Yes	No	No	sn	Unavailable
[14] (2011)	No	No	Yes	No	snb	Unavailable
[15] (2012)	Yes	No	No	No	sn	Unavailable
[9] (2012)	Yes	Yes	No	No	snbc	Unavailable
[16] (2012)	No	No	Yes	Yes	snb	Free
[17] (2013)	No	No	Yes	No	snbc	Free
[10] (2013)	Yes	Yes	Yes	No	i	Free
HCS-Neurons	Yes	Yes	Yes	Yes	snbci	Free

^1^Type of features: s = Soma-related, n = Neurite-related, b = Branching-related, c = Number of neuron, i = Generic image descriptor

Table 1 lists the functions of major methodologies published since 2010. The neurite-related features such as neurite length are most frequently used for quantifying the neuromorphology changes in specific cell culture. The soma-related features such as soma area are at rank 2, and the branch-related features such as branch complexity are at rank 3. The quantification analysis for single-neuron phenotypic changes is successfully demonstrated in the studies [11-17]. In additional, classification analysis was implemented in the studies [14,16,17] and regression analysis is also proposed in the work [17]. For analyzing HCS-based multi-neuron images [3-10], automatic feature extraction is considered as an essential technique. The classification analysis was only applied in [10] apart from neuron feature descriptor (NFD), the generic feature descriptor (GFD) was verified to provide a promissing result [10]. Surprisingly, the regression analysis is out of attention in multi-neuron-image-based HCS.

In this study, we develop an automated analysis method with novel descriptors of neuromorphology features for analyzing HCS-based multi-neuron images, called HCS-neurons. At first, we extend our previous work [3] to propose a neuron feature descriptor which consists of 13 features and is able to effectively quantify neuronal morphology changes. To make a comprehensive study on the collective phenotypic changes, we propose a generic feature descriptor consisting of several promising image features by utilizing pixel intensity, image moment, and texture information. The HCS-neurons method achieves the automatic feature extraction using an extended version of NeurphologyJ [3], feature analysis using ANOVA analysis and regression analysis, feature selection using an optimization approach based on an inheritable bi-objective combinatorial genetic algorithm (IBCGA) [18], classifier design based on support vector machine (SVM) [19] with the selected features.

To evaluate HCS-neurons, we treated P19 neurons with nocodazole (a known microtubule depolymerizing drug) at six concentrations ranging from 0 to 1000 ng/mL. The multi-neuron images treated using 6 different nocodazole drug concentrations (NDC) were selected as our benchmark because nocodazole has a well-known ability to directly affect neurite morphology [20-23]. The identified phenotypic changes of neurites were consistent with the known effect of nocodazole in promoting neurite retraction. Three identified features, total neurite length, average neurite length, and average neurite area can achieve an independent accuracy as high as 90.28% for the six-dosage classification problem.

## Methods

The proposed HCS-neurons method using the quantification and classification strategies for HCS of multiple neuron phenotypic changes response to 6 dosages of NDC is described. The multi-neuron images are preprocessed in the same way as reported in the previous work [3]. The binary images are generated for establishing the NFD and GFDs datasets. Additional gray-scaled images are necessary for some GFD features. We perform standard statistical analyses using ANOVA and regression for the NFD features which can directly show easily interpretable changes in neuronal morphology. To identify the descriptors which correlated to phenotypic changes upon NDC variations, the SVM-based classification analysis was used to evaluate both datasets. Finally, the IBCGA method was applied to select a small set of features by optimizing SVM prediction performance.

### Dataset

Nocodazole is a known microtubule depolymerizing drug which can lead to impaired neurite development. The image acquisition procedure used here is the same as described in [3]. For self-completeness, the procedure is concisely described below. Embryonic carcinoma P19 cells were maintained at 37°C in 5% CO_2 _in minimum essential medium supplemented with 2 mM glutamine, 1 mM sodium pyruvate, and 10% (v/v) fetal bovine serum. The drug experiment was performed on 96-well plates. Each well on the plate was pre-spotted with 800 ng of proneural gene (Ascl1) expressing plasmid and 0.4 μL of Lipofectamine 2000 in a total of 50 μL serum-free minimum essential medium. After 20 minutes, 16 000 P19 cells in differentiation medium (minimum essential medium supplemented with 2 mM glutamine, 1 mM pyruvate, 5% fetal bovine serum) were added to each well and maintain in a 37°C, 5% CO_2 _incubator. 72 hours post-transfection, P19 cell cultures were treated with DMSO (control) and various concentrations of nocodazole (10, 50, 100, 200, and 1000 nM). After 24 hours of incubation, drug-treated cells were fixed with 3.6% formaldehyde in PBS. Fluorescence images were acquired with an Olympus IX-71 inverted microscope equipped with a CoolLED fluorescent light source (400 nm and 490 nm wavelength modules) and a Hamamatsu ORCA-R2 camera (6.45 μm × 6.45 μm pixel dimensions). Chroma BFP-A-Basic and Olympus U-MWIBA3 filter sets were used to image DAPI and DyLight488 fluorophores, respectively. Olympus Plan Apochromat objective lenses (10x 0.4 N.A. or 60x 1.35 N.A.) were used to collect the images.

**Figure 1:**
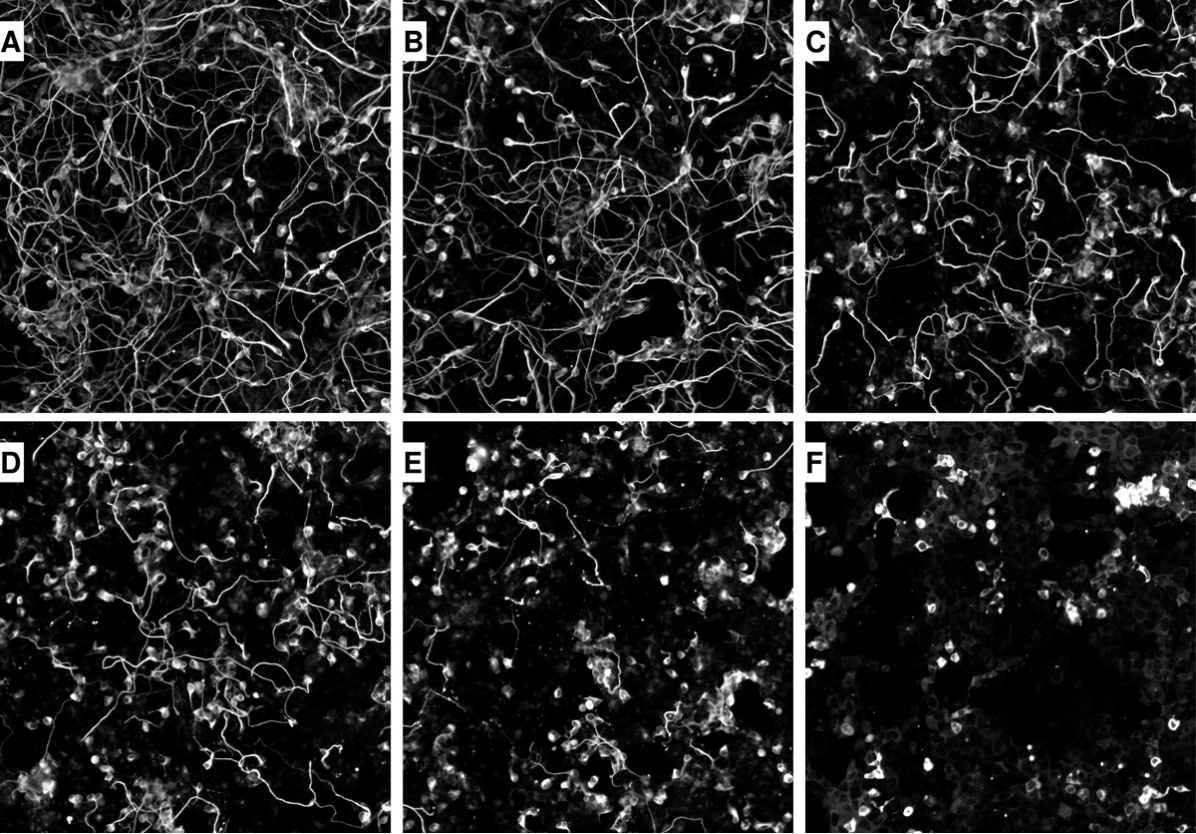
**Examples of neuron images with increasing Nocodazole concentrations [0,10,50,100,200,1000] (ng/mL) in order**.

A total of 216 images called Noco216 were analyzed to examine the morphological adaptation of neuron cells to the amount of drug. Images were divided into 6 classes (each class had 36 images) based on the dosage of nocodazole applied. These 216 images were all multi-neuron images since they contained hundreds of neurons. Both ANOVA analysis and regression analysis are based on NFD extracted from images in this dataset. To define the classification problem, we implemented a stratified random sampling to separate 2/3 of the image dataset as a training set called Noco144 and 1/3 of image dataset as a testing set called Noco72. The summary of these datasets is listed in Table 2. Typical neuron images from each class (i.e. each different nocodazole concentration) are shown in Figure 1. It is readily observable that neuronal morphology exhibited dramatic difference at different dosage of nocodazole.

**Table 2:** Summary of all datasets in this research separated by six-dosage of Nocodazole concentration

*Concentration (ng/mL)*	*0*	*10*	*50*	*100*	*200*	*1000*	*Total*
Original	36	36	36	36	36	36	216
Noco144	24	24	24	24	24	24	144
Noco72	12	12	12	12	12	12	72

### Neuron Feature Descriptor (NFD)

The standard multi-neuron image descriptors from NeurphologyJ [3] were used which include somaCount, somaArea, neuriteLength, neuriteArea, attachmentPoint# and endingPoint#. The features somaCount and somaArea are the numbers of soma and summation of all soma area in a multi-neuron image, respectively. The features neuriteLength and neuriteArea represent the total length of all neurites combined together and summation of the entire area covered by all somata in an image, respectively. The feature attachmentPoint# describes the total number of all neurite attachment point where neurites connect to a soma appeared in an image. The feature endPoint# describes the total number of all neurite end point appeared in an image. In addition, we developed an additional further called branchPoint# which is determined by using a 3x3 mask consisting of all possible 3x3 branch patterns. Each 1-pixel-wide neurite was automatically matched to one of these patterns to characterize the branch points.

Features measuring the cumulative value of all cells (e.g. total length of all neurites), are sensitive to cell counts, e.g. a reduced cell density will change the phenotypic readout even when cells are not treated with drugs. Therefore, the second part of NFD includes Avg_somaArea, Avg_neuriteLength, Avg_neuriteArea, Avg_attachmentPoint#, Avg_endingPoint#, and Avg_branchPoint# which were computed by dividing all of the standard features (except somaCount) by somaCount. The standard descriptors describe global changes of multiple-neuron neuromorphology patterns whereas the normalized values approximate a local descriptor for each single neuron in the multi-neuron image. Summaries of the analyzed descriptors are listed in Table 3.

**Table 3:** Description of the neuron feature descriptor extended from NeurphologyJ [3].

*Feature Name*	*Description*	*Extension*
somaCount	The total number of somata.	No
somaArea	The total area of somata in pixel.	No
neuriteLength	The total length of all neurites in pixel.	No
neuriteArea	The total area of neurites in pixel.	No
attachmentPoint#	The total number of attachment points.	No
endingPoint#	The total number of ending points.	No
branchPoint#	The total number of branching points.	Yes
Avg_somaArea	Avg_somaArea = somaArea/somaCount.	Yes
Avg_neuriteLength	Avg_neuriteLength = neuriteLength/somaCount.	Yes
Avg_neutiteArea	Avg_neutiteArea = neuriteArea/somaCount.	Yes
Avg_attachmentPoint#	Avg_attachmentPoint# = attachmentPoint#/somaCount.	Yes
Avg_endingPoint#	Avg_endingPoint# = endingPoint#/somaCount.	Yes
Avg_branchPoint#	Avg_branchPoint# = branchPoint#/somaCount.	Yes

### Generic Feature Descriptor (GFD)

The Generic feature descriptor (GFD) consists of several well-known image descriptors which have been used to classify images in many applications and is included in HCS bioimage tools [24-27]. A summary of these features including references and parameters is given in Table 4. However, these descriptors are not popular in the research of neural image analysis (see Table 1) and have never been proposed as tools to investigate neuronal phenotypic changes. Part of the reason may be due to difficulty of implementation. For example, contour-based descriptors such as the fourier transform and bending energy [28] require the development of neuronal contours which is a difficult task due to the intersection of spanning arbors of neurons. These contours then require additional single-neuron segmentation to process the multi-neuron images. Thus, in this study, we focused on methods that could easily be implemented without the prerequisite of the segmentation process and readily describe characteristic of multiple-neuron neuromorphology.

**Table 4:** Description of generic feature descriptors and their related references

*Descriptor Name*	*Number of Features*	*Type of Feature*	*Parameter Setup*	*Reference *
Zernike Moment	4,9,25,81	B/W	2^nd^, 4^th^, 8^th ^,16^th ^moment orders.	[29]
Legendre Moment	9,25,81,289	B/W	2^nd^, 4^th^, 8^th ^,16^th ^moment orders.	[30]
Tchebichef Moment	9,25,81,289	B/W	2^nd^, 4^th^, 8^th ^,16^th ^moment orders.	[31]
Generic Fourier	60	B/W	5 angles and 12 frequencies.	[32]
Haralick Texture	180	Gray-Scaled	18 Haralick texture measurements from 8-bins gray-levels coocurence matrix (GLCM) created from 1 to 5 pixel distances with 0, 45, 90, 135 angles.	[33,45]
Gabor	60	Gray-Scaled	6 levels of orientation and 5 levels of scaling.	[34]
Daubechies4	30	Gray-Scaled	10 level wavelet decomposition	[34]
NeurphologyJ	13	B/W	Contrast = 13, Soma intensity = 288, Neurite width = 5 and Particle cleanup = 15	[3]

Effective shape descriptors based on orthogonal polynomial moments include Zernike Moment [29], Legendre Moment [30] and Tchebichef Moment [31] which are extracted with respect to 2^nd^, 4^th^, 8^th ^and 16^th ^moment orders. In additional to moment based descriptors, the Generic Fourier method [32] which is another shape descriptor based on the Fourier transform of polar coordinates has also been implemented. All shape-based GFDs are calculated using binary images generated from the preprocessing procedure of NeurphologyJ.

Additional outstanding texture-based descriptors consisting of Haralick, Gabor and Daubechies are also designed. The Haralick descriptor is based on a spatial relationship evaluated from a gray level co-occurrence matrix (GLCM) of gray-scaled images [33]. In [34] Gabor and Daubechies successfully used this feature to predict protein location. The Gabor descriptor uses a mean and standard deviation of gray-scaled image convoluted by the Gabor filter [34]. Daubechies is an averaged energy value calculated from the 10 levels of wavelet decomposition of gray-scaled image using the Daubechies4 wavelet function [34].

### Statistical analysis - ANOVA and regression

The six sets of 36 multi-neuron images generated using different nocodazole drug concentrations were statistically analyzed using 1-way ANOVA and regression analyses to determine whether there were any statistically significant differences amongst different drug levels for each of the thirteen tested features. The statistical analysis is performed using the SAS/STAT software, Version 4.3 (4.3.0.12251) of the SAS System for Windows.

Each of the 13 features was tested for normality and equal variances (homoscedasticity) using the Bartlett's test in SAS/STAT. Those features which were found to satisfy the assumptions of the standard 1-way ANOVA were then analyzed with a post-hoc test using the Tukey honestly significant difference (HSD) test. Those features that were found to be heteroscedastic with significant differences between the within group variances were tested in SAS with the Welch's variance-weighted ANOVA before then being further analyzed with the Tukey HSD post-hoc test.

Those features which were found to have significant variation between groups were then further tested with regression analyses. For each of these features, linear and quadratic regression models were tested to determine the best fit for the changing Nocodazole concentration relationship. The linear model was chosen if there was no significant improvement with the addition of the quadratic term [35] otherwise the quadratic regression model was chosen. Briefly, the amount of between group variations not explained by the linear model is compared to the increase in explained variation found using the quadratic model. If this increase is more than 5% of the total not explained remaining variations then the quadratic model provides a significantly improved fit to the data. In all cases the total between group variation explained by the optimal regression model was then compared to the total between group variation from the ANOVA. If the regression model explained greater than 90% of the total variation, we considered it a successful model.

### Inheritable bi-objective genetic algorithm

The bi-objective 0/1 combinatorial optimization problem for feature selection has two objectives: minimizing the number of selected informative features and maximizing classification accuracy. The inheritable bi-objective combinatorial genetic algorithm (IBCGA) is an efficient feature selection method based an intelligent genetic algorithm (IGA) [36]. To efficiently solve the combinatorial optimization problem *C(n,m) *where n is number of candidate features, IGA uses an intelligent crossover operation based on orthogonal experiment designed to efficiently explore the possible solution of the combinatorial problem. IBCGA can efficiently explore the possible solutions to *C(n, r±1) *by inheriting a good solution to *C(n, r) *[18]. This inheritable mechanism also allows to economically choose the feature set for improving predication accuracy.

The IBCGA encodes features in the descriptors as binary genes for feature selection and encodes parameters of support vector machine (SVM) in using the IGA chromosome. The IGA chromosome consists of n features bits (n is feature number) and two 4-bit IGA-genes to tune the parameters C and γ of SVM. One feature is included in the SVM classifier if the encoded value for the gene is equal to 1. For tuning the SVM parameters, the 16 encoded values of γ and C are belonging to {2^-7^, 2^-6^, ..., 2^7^, 2^8^}.

The IBCGA procedure can be briefly summarized as follows [18]:

Step1) Randomly generate an initial population of individuals using r = R_start_. (Initialization)

Step2) Evaluate the fitness values of all individuals using the fitness function which is the 10-fold cross-validation (10-CV) classification accuracy of using SVM. (Evaluation)

Step3) Form a mating pool using tournament selection. (Selection)

Step4) Perform orthogonal array crossover on the selected parents. (Crossover)

Step5) Apply the swap mutation operator to the randomly selected genes in the new population and increase the number of generations. (Mutation)

Step6) Stop if the number of generations is equal to 20; otherwise go to Step 2). (Termination)

Step7) If r > R_end_, randomly change one bit in the binary IGA-genes for each individual from 0 to 1; decrease the number r by one, go to step2); otherwise stop the algorithm. (Inheritance)

In this study, the size of the candidate feature set selected by the IBCGA was ranged from R_start _= 13 to R_end _= 1 corresponding to number of features in NFD. Eventually, the best solution in terms of 10-CV classification accuracy is selected as a final solution.

### Performance evaluation of multi-neuron image descriptor

We hypothesize that the phenotypic changes in multiple neuron systems can be observed in two general ways consisting of neural dependent and neural independent mechanisms via NFD and GFDs, respectively. The classification approach is familiarly applied as an important tool for characterizing each phenotypic change of a single neuron [37-39]. Thus, the suitable multi-neuron image descriptor that is highly correlated to the variation of 6 dosages of NDC must be efficient to use as a training set for constructing the classifier for multi-neuron image input. The performance comparison between NFD and GFDs is evaluated by the prediction accuracy of independent test. Moreover, the discrimination power of utilizing single morphology and multiple morphologies as the classifiers are also examined. We used SVM which is a well-known efficient classification method for our multi-neuron image classification because of its successful use in numerous image classification studies [40-44]. The wide spread usage of the LibSVM tool also encouraged us to utilize it in all of our experiments [19]. An SVM with a radial basis function kernel was used to create the classifier using Noco144 as a training set. A grid search technique was then used to select for the proper values of the SVM parameters while maximizing the classification accuracy of 10-CV. Then, the Noco72 data set was used as an independent test set to assess the generated classifier performance.

## Results

### Images preprocessing

Our original raw images were gray-scaled taken using fluorescence microscopy. We applied a preprocessing technique in the same way as reported previously in NeurphologyJ [3]. The four input parameters consisting of contrast, soma intensity, neurite width and particle cleanup value were set to 13, 288, 5 and 15, respectively. Examples of the original images from the six classes are shown in Figure 1.

**Figure 2:**
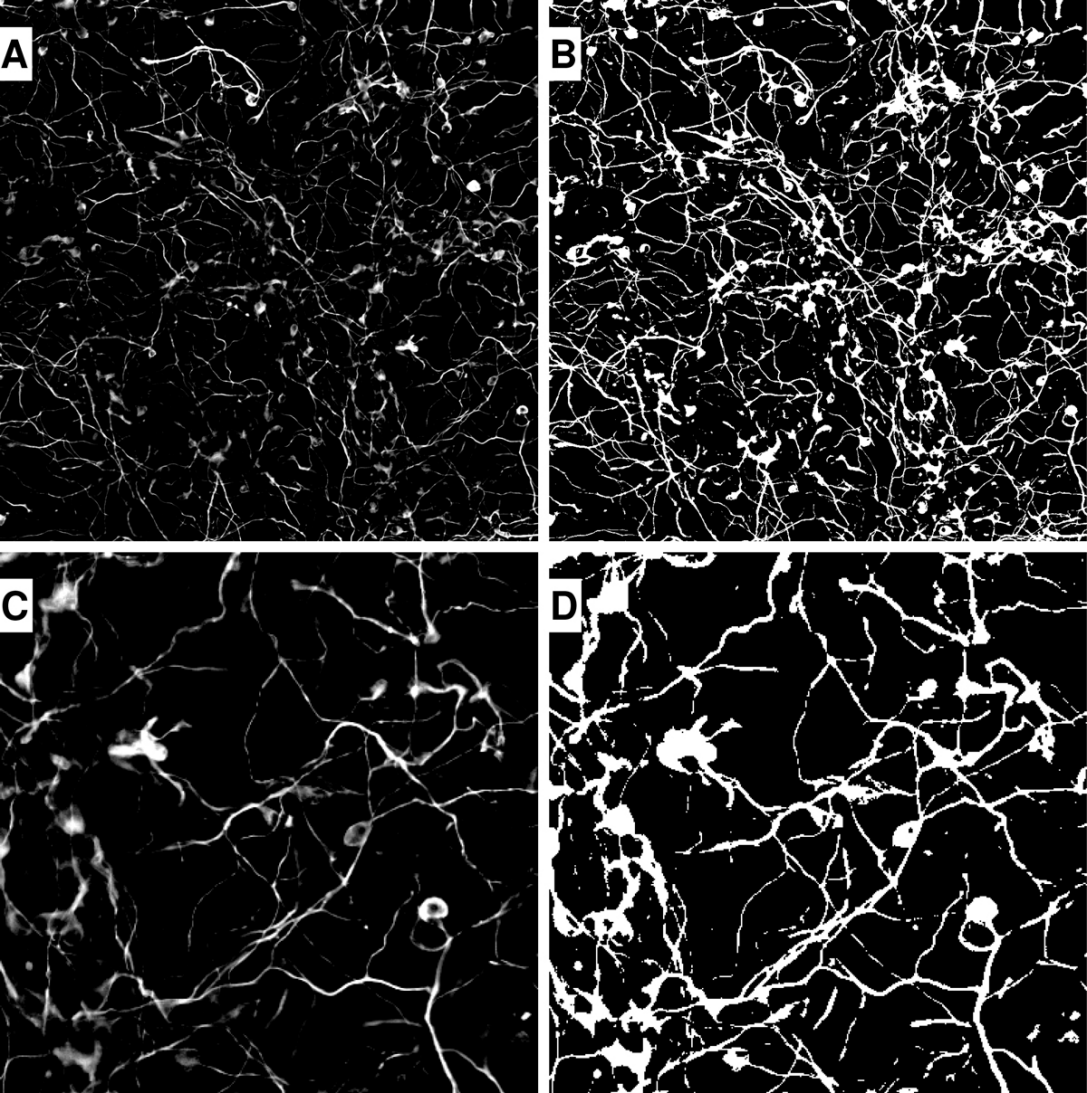
**Example of preprocessed neuron images**. (a) background removed gray-scaled image (full), (b) black and white image (full), (c) background removed gray-scaled image (zoom), and (d) black and white image (zoom).

After background removal and isolated object elimination, the resulting gray-scaled images of class 1 representing the wild type are displayed in full size (Figure 2a) and 5x zoom (Figure 2c). The binary image is obtained by NeurophologyJ and displayed in full size (Figure 2b) and 5x zoom (Figure 2d). The binary images illustrate the complexity of the analysis due to multiple intersections of neurites from different neurons. These images also show the key point of our software design. We focused on analyzing all neurons and their neurites in the entire image instead of spending a huge amount of time on analyzing individual neurons one by one.

### Multi-neuron images feature extraction

**Figure 3:**
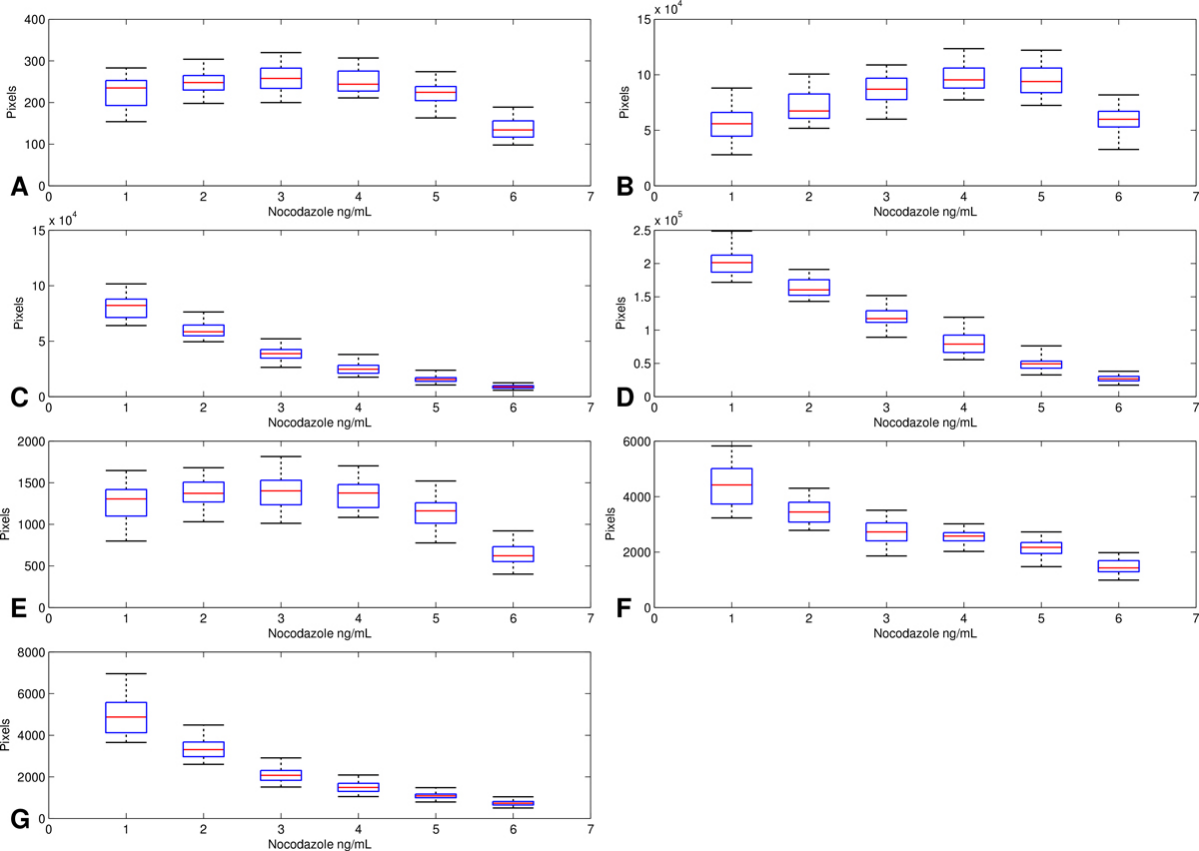
**Box plot displaying relationship between Nocodazole concentration and some neuron features**. (a) somaCount, (b) somaArea, (c) neuriteLength, (d) neuriteArea, (e) attachmentPoint#, (f) endPoint# and (g) branchPoint#.

**Figure 4:**
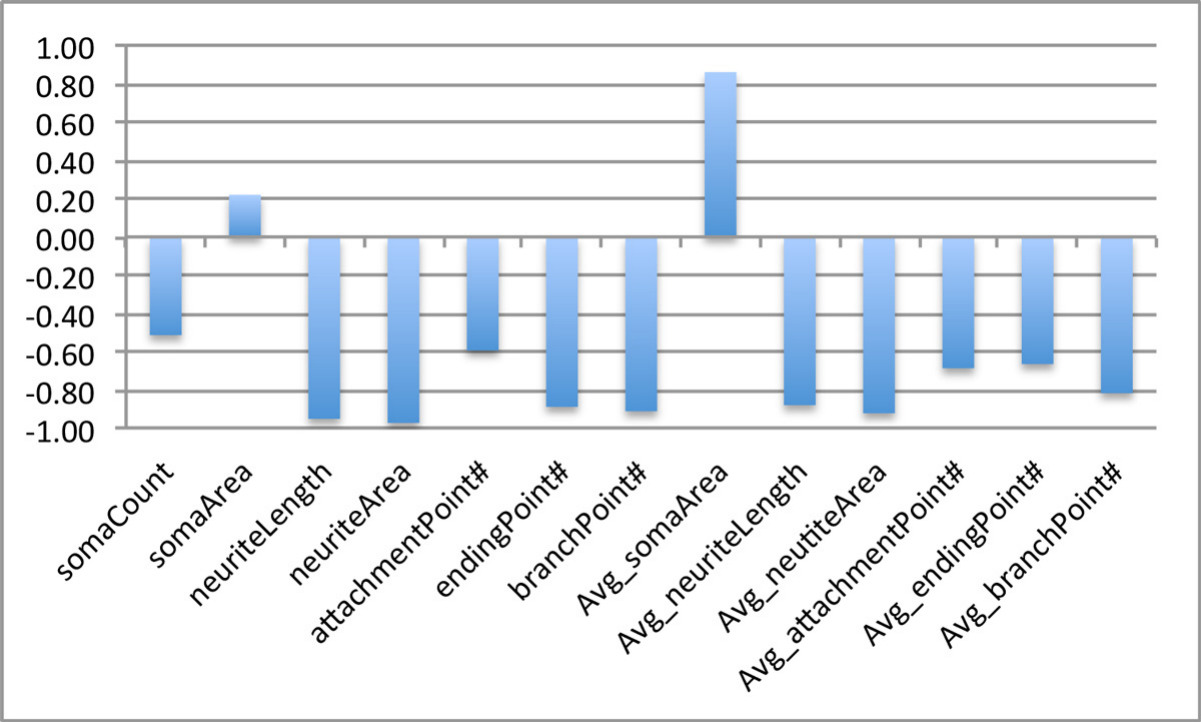
**Pearson's correlation between the numerical value of each neuron feature and nocodazole concentration**.

The crucial NFD features consisting of NeurphologyJ features and branchPoint# extracted from all images are illustrated using the box plots shown in Figure 3. From Figure 3, all the distributions of neurite-related features comprising neuriteLength (Figure 3c), neuriteArea (Figure 3d), endPoint (Figure 3f) and branchPoint (Figure 3g) have similar trends as the concentration of nocodazole increased. Notably, neuriteLength and neuriteArea exhibited the identical trend, this suggest the thickness of the neurite (which equals neuriteArea divided by neuriteLength) was not affected by increasing concentration of nocodazole. In contrast, the distributions of soma-related features comprising somaCount (Figure 3a), somaArea (Figure 3b) and attachmentPoint (Figure 3e) stay almost constant throughout various nocodazole concentrations. Pearson's correlation analysis between various NFD features and nocodazole concentration (NDC) are shown in Figure 4. This result shows that most of the NFD especially neurite-related features have an inverse relation to increasing NDC. Remarkably, the soma area related features tend to have a correlated relationship with NDC. These results provide some insight into the relationships between neuronal morphology and nocodazole concentration. However, to obtain the quantitative relationship between them, stronger statistical tools such as ANOVA and regression analysis are required and described below. For GFDs, only classification analysis is conducted.

### ANOVA analysis of NDC affect to NFD

Each of the 13 features was tested for normality and equal variances (homoscedasticity) using the Bartlett's test in SAS/STAT. Of the 13 features, only somaCount and somaArea were found that they do not have significant difference within group variances or depart from normality. These two features were therefore tested with the standard homoscedastic ANOVA analysis to detect the presence of significant differences between the group means and were then post-hoc tested using the Tukey honestly significant difference (HSD) test with SAS/STAT.

The other 11 features were found to have significant differences between the within group variances and therefore were tested in SAS with the Welch's variance-weighted ANOVA before being tested with a post-hoc test using the Tukey HSD test. The mean values of each nocodazole concentration for each of the 13 features are shown in Table 5 with the differences between the group means shown by way of the standard superscript letters designating means which do not differ at the 95% significance level. The second column of Table 5 displays the ANOVA significance R^2 ^values which for 215 total degrees of freedom is significant if R^2 ^> 11.2%. In particular, the soma count which was used to normalize six of the other features exhibited a significant increase in the group means for the 50 ng/mL concentration over the untreated soma count. This feature then showed significant decreases in the soma count for further increasing nocodazole concentrations up to 1000 ng/mL. The soma area values had a similar appearing quadratic relationship for increasing concentrations of nocodazole with an initial increase in area values for concentrations up to 50 ng/mL and then a significant decrease for the 1000 ng/mL concentration back to untreated levels. Only these two soma features and the attachment point number feature exhibited obvious quadratic relationships, whereas the other 10 features generally seemed to uniformly increase or decrease with increasing nocodazole concentrations.

**Table 5:** Anova analysis of the effect of nocodazole concentration on the neuron feature descriptor.

*Feature* *Name*	*ANOVA* *R^2 ^(%)*	*Nocodazole concentration (ng/mL)*						*Scale* *(pixels)*
			
		0	10	50	100	200	1000	
somaCount	65.9	224^A^	249^B^	255^B^	251^B^	221^A^	137	x1
somaArea	62.3	568^A^	716^B^	867^C^	970^D^	949^CD^	591^A^	x10^2^
neurite Length	94.4	808	598	385	251	156	89	x10^2^
neuriteArea	95.3	203	164	119	80	49	27	x10^3^
attachmentPoint#	69.5	126^AB^	138^BC^	140^C^	136^BC^	115^A^	64	x10
endingPoint#	82.5	440	345	270^A^	255^A^	216	147	x10
branchPoint#	91.0	493	336	209	152	109	75	x10^2^
Avg_somaArea	76.8	250	287	340	388	431^A^	432^A^	x1
Avg_neurite Length	88.0	372	244	151	100	71^A^	65^A^	x1
Avg_neurite Area	91.3	924	666	470	317	222^A^	198^A^	x1
Avg_attachment Point#	58.4	562^A^	554^AB^	546^AB^	542^B^	520	468	x10^-2^
Avg_ending Point#	68.6	203	141	106^A^	102^A^	98^A^	108^A^	x10^-1^
Avg_branch Point#	85.1	227	137	82	60^A^	50^A^	55^A^	x10^-1^

Values with the same superscript letter are not different at the 5% experiment-wise level. Values with no superscript are different from all other values for that feature. The R^2 ^ANOVA value is the fraction of variation that occurs between groups versus the total variation for that feature. For 216 data measurements values of R^2 ^over 11.2% show a significant difference between group means.

For nine of these features, the means significantly decreased with increasing nocodazole concentrations. Eight of these features were pairs of the normal and average features: neurite length, neurite area, ending point number and branch point number. The last one feature with monotonically decreasing average values was the average attachment point number. The reduction in neurite length correlates nicely with the known effect of nocodazole in causing neurite retraction [23]. The decreasing amount of branch points and end points with increasing nocodazole concentration also indicate a nocodazole-dependent degradation in neurite complexity. The reduction in average neurite area with increasing nocodazole concentration also shows the further effect of neurite retraction on the morphological properties of neurites.

The last feature showed an increase in average soma area while the nocodazole concentration was increasing. After several images were manually inspected, we found that HCS-Neurons presumed that these somata are regarded as a single soma. The scenario reveals that the somata from several neurons tend to cluster together at high nocodazole concentration.

### Regression analysis of NDC affect to NFD

In the ANOVA analyses described above, significant differences were found for each of the 13 features with the observed patterns showing generally linear changes with respect to NDC with a few obvious cases showing a quadratic relationship. Therefore we chose the linear and quadratic regression models to find the optimal relationships between increasing nocodazole concentrations and each feature.

To assess the quality of each model we determined the R^2 ^values for the amount of between group variations explained by the two regression models. As is well known, the R^2 ^values for quadratic regression will always be higher than those for linear regression models, so we tested whether the improvement in the quadratic model R^2 ^value was significantly above that of the linear model [35]. Table 6 summarizes these quantities for each of the 13 features with the first column showing the total between group variations for each feature (these R^2 ^values are also shown in Table 5 but they are duplicated here for ease of interpretation). The next two columns display the R^2 ^values for the linear and quadratic regression models respectively. The fourth column in Table 6 shows how much of the overall between group variation is accounted for by the optimal regression model. The optimal regression models explain over 95% up to 99.7% for every feature except Soma area which only had 72.7% of the between group variation explained by the quadratic model. This column shows that the optimal regression models for increasing NDC values are very well described by simple linear and quadratic models. For the soma area we did try fitting the data to a third order polynomial which yielded an R^2 ^value of 61.5% and accounted for 98.7 of the between group variation. The shape of the third order polynomial looked very quadratic with a local minimum near zero and a sharp maximum near NDC values around 170 ng/mL. The final column in Table 6 shows the regression equation for the optimal linear or quadratic model which is also shown in bold in the appropriate regression model R^2 ^column.

**Table 6:** Regression analysis of the effect of Nocodazole concentration on the neuron feature descriptor.

*Feature Name*	*ANOVA* *R^2 ^(%)*	*Linear* *Regression* *R^2 ^(%)*	*Quadratic Regression* *R^2 ^(%)*	*Variance explained* *(%)*	*Regression Equation*
somaCount	65.9	18.6	**62.7**	95.1	- 35.3x^2 ^+ 80.4x + 219.3
somaArea	62.3	7.3	**45.3**	72.7	13400x^2 ^+ 45000x + 5200
neurite Length	94.4	**91.5**	91.7	96.9	- 26100x + 81800
neuriteArea	95.3	**91.2**	91.6	95.6	- 63500x + 213000
attachment Point#	69.5	26.6	**66.7**	95.9	- 212x^2 ^+ 449x + 1230
endingPoint#	82.5	**82.3**	82.3	99.7	- 976x + 4420
branchPoint#	91.0	88.1	**89.7**	96.8	203x^2 ^+ 2080x + 5030
Avg_soma Area	76.8	**70.4**	70.4	91.6	69.4x + 238
Avg_neurite Length	88.0	82.8	**86.1**	97.8	22.4x^2 ^- 177x + 381
Avg_neurite Area	91.3	87.3	**88.3**	95.6	29.7x^2 ^- 352x + 946
Avg_attachment Point#	58.4	42.3	**56.9**	97.4	- 0.167x^2 ^+ 0.207x + 5.60
Avg_ending Point#	68.6	54.2	**68.0**	99.1	1.77x^2 ^- 8.62x + 20.5
Avg_branch Point#	85.1	75.7	**84.0**	98.7	2.10x^2 ^- 12.4x + 23.1

The R^2 ^ANOVA column shows the total amount of between group variation for each feature. The next two columns show how much of the total variation is explained by a linear model or a quadratic model. If the quadratic model explains more than 5% of the remaining between group variation over the linear model that model is chosen otherwise the quadratic model doesn't improve the fit significantly enough to justify the additional complexity. Essentially, if using the quadratic model increases the R^2 ^fit by more than 0.5% over the linear model we choose that one [35] The variance explained column shows the total percentage of the between group variation explained by the chosen model. The final column shows the equation for the best fit model for each feature.

#### Regression analysis of nocodazole drug concentrations on soma count

**Figure 5:**
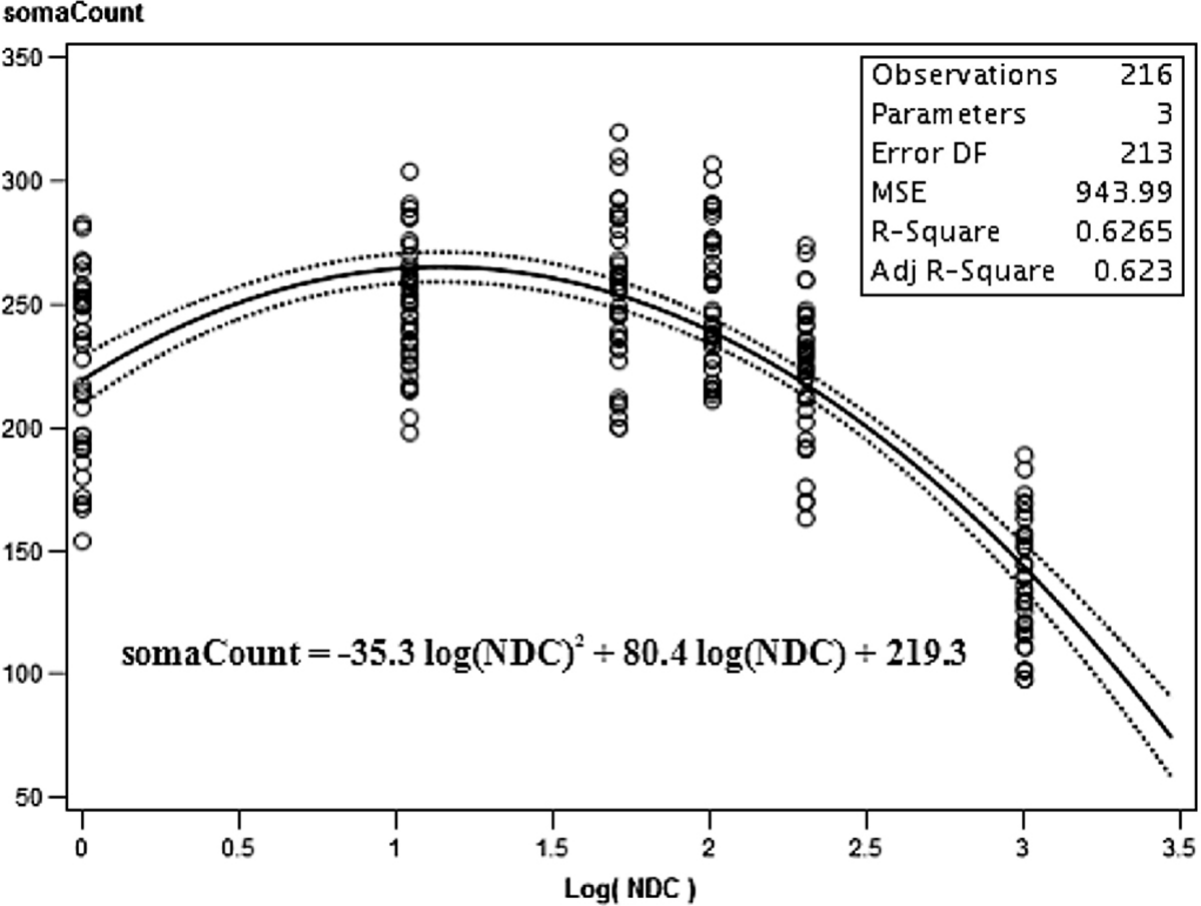
**The quadratic relationship between somaCount and the logarithm of nocodazole concentration**. The variation described by this regression accounts for 95.1% of all of the between group variation for somaCount.

Since a very strong effect was found for nocodazole drug concentration on somaCount, and that the value of somaCount showed a strong quadratic relationship with increasing NDC up to 50 ng/mL and then decreased with further NDC increases the fact that the optimal quadratic regression model explained 95.1% of the between group variation was not unexpected. The results of this regression analysis are shown in Figure 5 where the best fit line is shown in black and the 95% confidence intervals of the mean values are shown as dotted lines. This regression analysis found that R^2 ^= 62.7% of the variation for this feature was described by the line Y = (-35.3 ± 2.2)X^2 ^+ (80.4 ± 6.9)X + (219.3 ± 5.0) where Y is the somaCount value and × is the Log(NDC) value. In terms of the total variation described by this best fit line, (62.7/65.9) = 95.1% of all between group variation is accounted for by this quadratic regression line. The somaCount residuals were also found to not vary significantly from a normal distribution (p = 0.085). The feature somaCount satisfied all of the assumptions of both 1-way ANOVA and regression and was very well described by the quadratic dependence model on Log(NDC). These results are summarized in Table 6 which also shows the quadratic model R^2 ^value, the quadratic equation and the percent of between group variation explained by the optimal quadratic model.

### Classification analysis

#### Performance comparison of each individual NFD feature

We separately applied each of the 13 specified features to evaluate the SVM prediction results from Noco72 by using Noco144 as a training data set. The optimal values of C and γ were then determined using a grid search. From the summary results listed in Table 7, neuriteLength and neuriteArea were found to be the most distinct features among six classes in this neuromorphology classification achieving identical 81.94% test accuracies with 86.80% and 84.72% training accuracies respectively. These results were followed by Avg_neuriteLength which had a 76.39% test accuracy and 77.08% training accuracy and Avg_neuriteArea which had a 73.61% test accuracy and 79.86% training accuracy. The feature branchPoint# was the last feature which scored dramatically above the rest of the features with a 72.22% test accuracy and 77.09% training accuracy.

**Table 7:** Classification performance of each feature in the **neuron feature descriptor**.

*Feature Name*	*10-CV Accuracy*	*Test Accuracy*
somaCount	43.06%	37.50%
somaArea	38.19%	34.72%
neuriteLength	86.80%	81.94%
neuriteArea	84.72%	81.94%
attachmentPoint#	40.97%	30.56%
endingPoint#	63.89%	52.78%
branchPoint#	77.09%	72.22%
Avg_somaArea	58.33%	47.22%
Avg_neuriteLength	77.08%	76.39%
Avg_neutiteArea	79.86%	73.61%
Avg_attachmentPoint#	44.44%	37.50%
Avg_endingPoint#	42.36%	40.28%
Avg_branchPoint#	68.75%	56.94%

Every soma-related feature showed a lack of discriminating power in this research. These results can be interpreted as showing that the nocodazole drug concentration affected both of the length and area of the neurites thereby achieving the highest accuracies. Figure 4 shows the Pearson's correlation value between each feature and the nocodazole drug concentrations. Most of the neurite-based features showed strong inverse relationship with nocodazole concentration, indicating that nocodazole exhibited strong negative effect on neurite development. According to the phenotypic changes of soma clustering, the increasing of nocodazole concentration also affected the average soma size since the feature Avg_somaArea had a correlation value of 0.86. We noted that this feature is also referred to as the averaged total area of adjacent somata described in previous section.

#### Performance comparison between NFD and GFD

The SVM analysis was used to run the descriptor assessment in the same way as described in the previous section. As shown in Table 8, our proposed multiple neuron descriptors and the Haralick texture descriptor gave the best predictive results. Both descriptors achieved 86.11% test accuracies. The second was the Gabor filter descriptor which achieved 81.94% test accuracies and the third was the Daubechies4 achieving 75.00% test accuracy. Remarkably, all GFDs using gray-scaled outperform the GFDs using binary information. For the group of GFDs using only the B/W images, the Zernike descriptor did the best achieving 70.83% test accuracies.

**Table 8:** Classification performance comparisons between the neuron feature descriptor and generic feature descriptors

*Feature Name*	*10-CV Accuracy*	*Testing Accuracy*
Zernike Moment	72.92%	70.83%
Legendre Moment	69.44%	62.50%
Tchebichef Moment	72.29%	62.50%
Generic Fourier	72.22%	55.55%
Haralick Texture	84.03%	86.11%
Gabor	78.47%	81.94%
Daubechies4	75.70%	75.00%
NeurphologyJ [3]	85.42%	80.56%
NFD (extended from NeurphologyJ [3])	84.72%	86.11%

Although our descriptor, the moment-based descriptors and the Generic Fourier descriptor utilize the same binary information to compute image features, the moment based descriptors and Generic Fourier descriptor cannot obtain high prediction accuracy because they were designed to handle shape information of single objects. In contrast, our dataset does not contain explicit shape information due to the many neurons distributed in each of the single images. Thus, all texture-based descriptors outperform the more restrictive explicit shape descriptors.

The Haralick descriptor is able to capture global information from each image including a measure of randomness, contrast and variance. This global approach is quite different from our multiple neuron based approach, but it does characterize global properties of the entire system which is a similar approach to our method and which enables it to achieve a high classification performance similar to our results. Furthermore, these efficient results of GFDs using gray-scaled information demonstrated the presence of intensity changes multi-neuron images according to NDC variations.

This result states that Haralick descriptor and NFD provide the same classification performance corresponding to phenotypic changes of neuron by varies NDC. However, the numbers of features in these two descriptors are hugely different, 180 and 13, respectively. Therefore, in conclusion, NFD is the best descriptor for phenotypic change of neuron due to its high classification performance and minimal number of features.

#### Optimal NFD features selected by IBCGA

**Figure 6:**
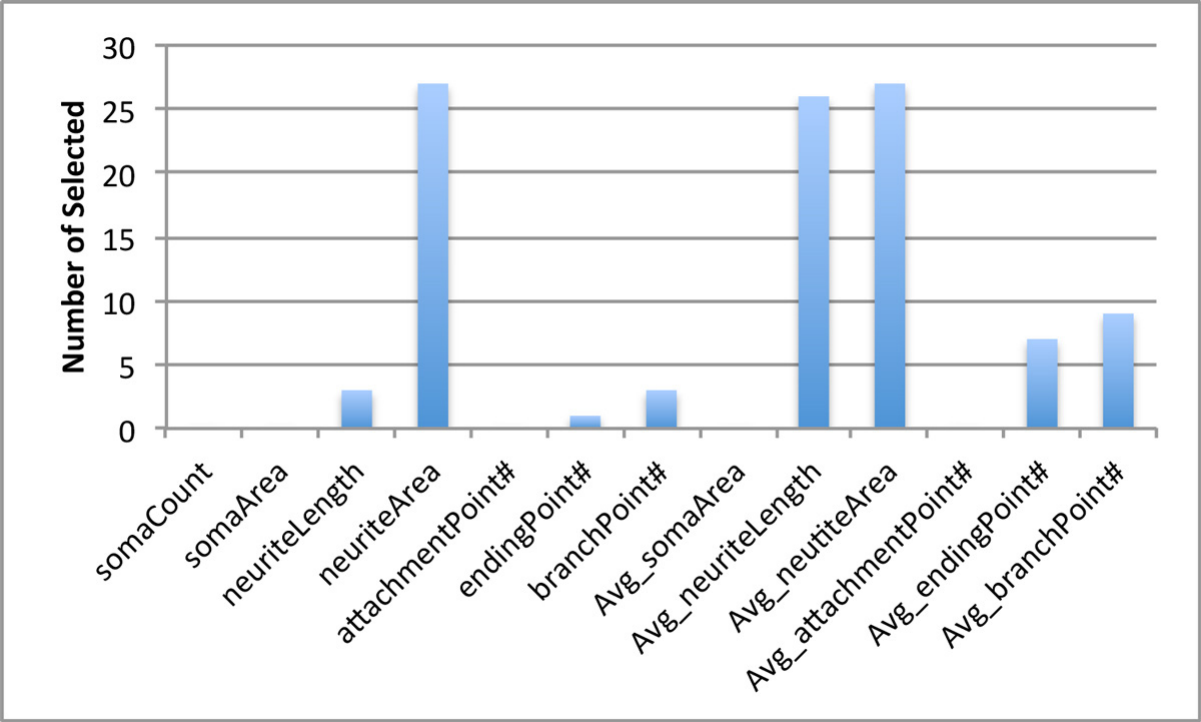
**Number of times of each feature was chosen by the IBCGA algorithm in 30 independent runs**.

According to the best choice in this HCS is NFD, IBCGA is applied to find the optimal solution to classify NDC using this descriptor. The 10-CV accuracy was evaluated using the IBCGA procedure to find suitable features and SVM parameters. This IBCGA procedure was executed 30 times to cope with the robustness problem of GA and produce 30 solutions. Figure 6 shows the number of times for each feature selected in the 30 independent runs. The features neuriteArea, Avg_neuriteArea and Avg_neuriteLength were selected with the largest number of times. The feature neuriteLength was not selected possibly due to redundant information with neuriteArea as mentioned in the previous section. This means either NeuriteArea or NeuriteLength can be substituted by each other. The solution with the highest prediction accuracy also contains these 3 features. Thus, the best SVM classifier is constructed by these 3 features with the SVM parameters, C = 2 and gamma = 16.

**Figure 7:**
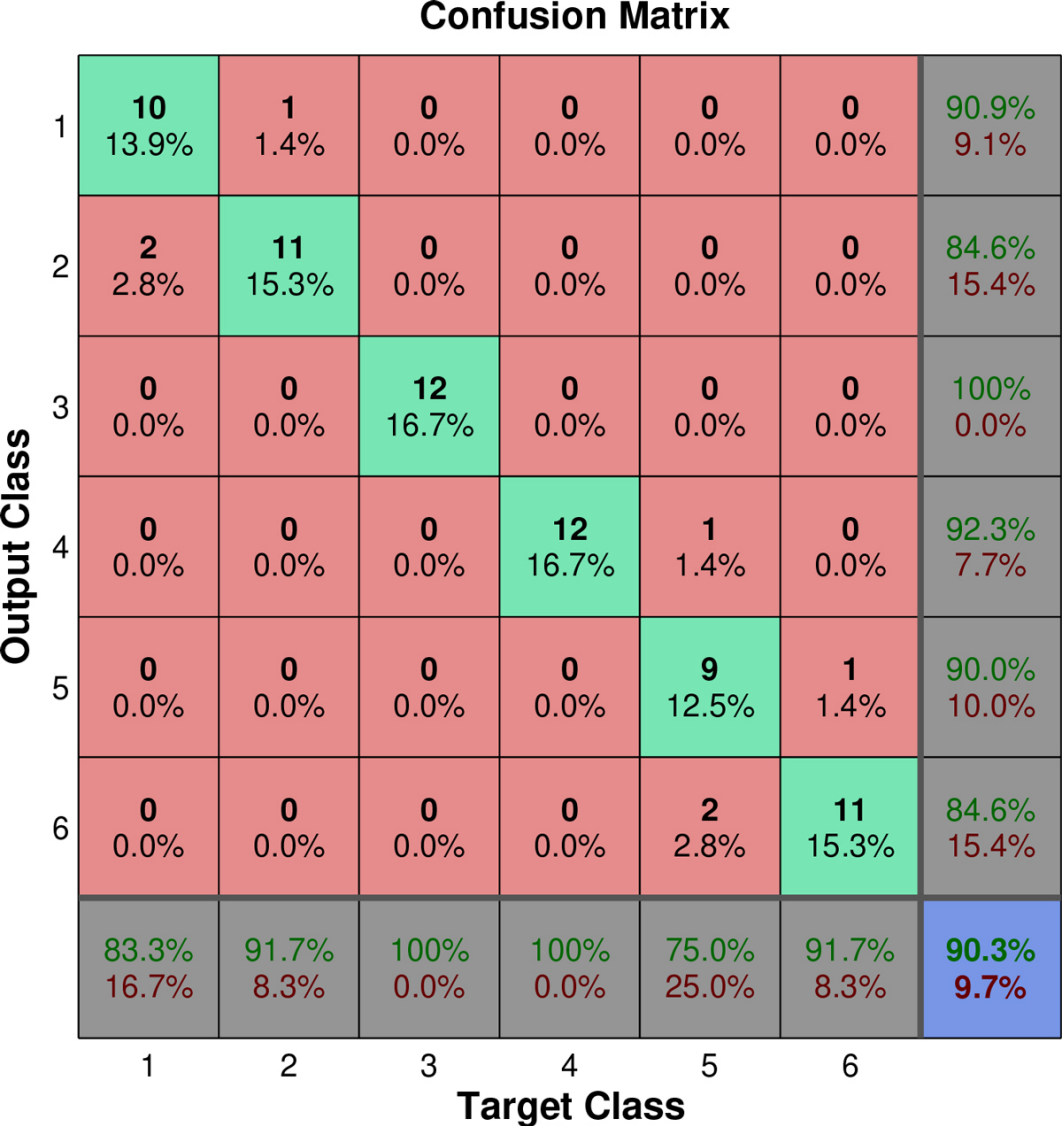
**Confusion matrix for the prediction results from the IBCGA features**.

This feature set was able to achieve accuracies of 90.28% and 91.67% for the test and training sets, respectively. The averaged performances for all of the solutions chosen by the IBCGA algorithm were 89.31% and 91.30% for the test and training sets, respectively. This result shows that the performance of the proposed multi-neuron image descriptor is stable with respect to the choice of feature set. To further evaluate the quality of our results, we constructed a confusion matrix from our prediction results which is shown in Figure 7. Interestingly, all of the locations where the prediction results were incorrect were located in the classes that were immediately adjacent to the correct class. This makes sense since our class definitions are based on increasing drug concentrations.

### Multiple neuron phenotypic changes response to NDC

The phenotypic transition of multi-neuron images detected by HCS-neurons is categorized into neuronal morphology alteration and pixel intensity alteration. In this section, the NFD describing neuronal morphology is in focus according to its optimal performance. We used the IBCGA method to separate out neuron-related features having the highest NDC discrimination power. The neuron morphology features identified by IBCGA were neuriteArea, Avg_neuriteLength and Avg_neuriteArea. The individual classification performances of these features were found to be more than 70%. From our regression analysis, we found significant decreases with increasing NDC levels for these 3 features. In particular, each of these features showed strongly decreasing values as NDC increased with reduced effect near the lower and upper NDC values. Although all three features showed essentially linear decreases with increasing log(NDC) values the strongest affects were for NDC values in the 50-200 ng/mL range.

In the previous section, the high correlation between Avg_somaArea and NDC was mentioned to have a simple linear relationship. The average soma area linearly increased with increased log(NDC) values while the average neurite area strongly decreased. While the inverse correlation between neurite length (or neurite area) and NDC is consistent with nocodazole's effect on inducing neurite retraction, the linear correlation between soma area and NDC has not been reported before. These data indicated that in addition to inducing neurite retraction, nocodazole might promote soma clustering at high concentration. It will be of great interest to examine this additional effect of nocodazole using other neurons.

For GFD morphology alteration, we found the strong evidence for intense differentiation according to NDC. However, detailed analysis to extract all of the interpretable information from these features can still be expanded. We plan to further explore the relationships between GFD and neuron development. In conclusion, there are many strong results from the statistical analysis and classification analysis for the interpretation of multi-neuron images. Therefore, the combination of these two approaches provides a powerful set of tools to generate useful information for neuroscientists to understand neuron modification response to drug treatment.

## Conclusions

In this paper we propose a complete high-content screening analysis method HCS-neuron for multiple neuron phynotypic modification response to different nocodazole concentration. Our extended version of multi-neuron image descriptor achieved prediction accuracies of 86.11%. We then used the IBCGA method to find the optimal feature set which resulted in an increase in prediction accuracy to 90.28%. The optimal set of features for this problem was found to be neuriteArea (Neurite Area), Avg_neuriteArea (Average Neurite Area) and Avg_neuriteLength (Average Neurite Length). Our quantification analysis also found that there were statistically significant changes in these descriptors which vary in exactly the way nocodazole is known to affect neurite growth. The intensity alteration also demonstrated by the high discrimination power of texture based generic descriptor i.e Haralick (86.11%), Gabor Filter (81.94%) and Daubechies4 wavelet decomposition (75%). However, the detail analysis is still hard to interpret at present.

The proposed HCS-neuron can extend HCS with single-neuron images to that with multi-neuron images and help improve the statistical significance of such results and leverage the strengths of high-throughput analysis to the understanding of neuron research. The proposed HCS-neurons is helpful to identify substances such as small compounds or RNAi molecules that can alter the morphological phenotype of an entire population of neurons using HCS. In addition to accomplishing this, our program and datasets are all available for download. Interestingly, we discovered a previously unknown effect of nocodazole on soma clustering. These results demonstrate effectiveness of the proposed quantitative analysis on the morpgological features from images containing multiple neurons.

The MatLab module of neurite feature descriptor (NFD) and image datasets are available at http://iclab.life.nctu.edu.tw/HCS-Neurons/.

## Competing interests

The authors declare that they have no competing interests.

## Authors' contributions

PC designed the system, implemented programs, carried out the analysis, and participated in manuscript preparation. EH provided biological knowledge, data sets and participated in the analysis. RC finished the statistical analysis and participated in manuscript preparation. HCL, LWK, HLH and SYH participated in the experimental design and carried out the analysis. HLH and SYH supervised the whole project, and participated in manuscript preparation. All authors have read and approved the final manuscript.

## Declarations

Publication of this article was funded by Ministry of Education, Taiwan, R.O.C.

This article has been published as part of *BMC Bioinformatics *Volume 14 Supplement 16, 2013: Twelfth International Conference on Bioinformatics (InCoB2013): Bioinformatics. The full contents of the supplement are available online at http://www.biomedcentral.com/bmcbioinformatics/supplements/14/S16.
